# Supplementary figures and images for: Promoting mechanism of serum amyloid a family expression in mouse intestinal epithelial cells
Source: PLoS One. 2022 Mar 18;17(3):e0264836. doi: 10.1371/journal.pone.0264836 (PMC8932556; doi:10.1371/journal.pone.0264836)

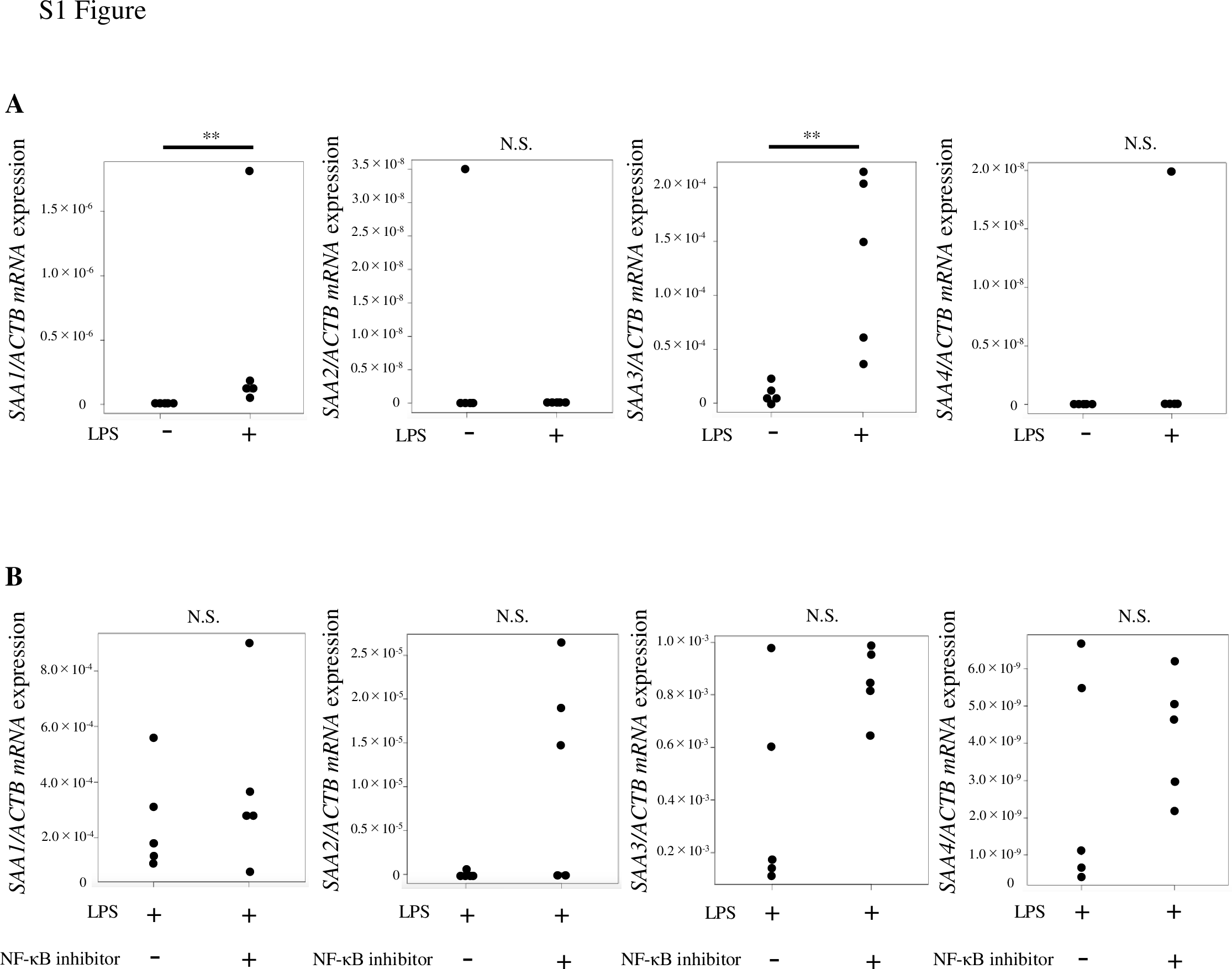

Supplement: S1 Fig — The addition of LPS significantly increases the expression level of SAA1/3 (A), an effect that is not inhibited by the NF-κB inhibitor, BAY11-7082 (B). The SAA expression level was normalized to that of ß-actin (ACTB) and is presented as the mean ± standard deviation (n = 5). **P < 0.01. (TIF) [file pone.0264836.s001.tif]

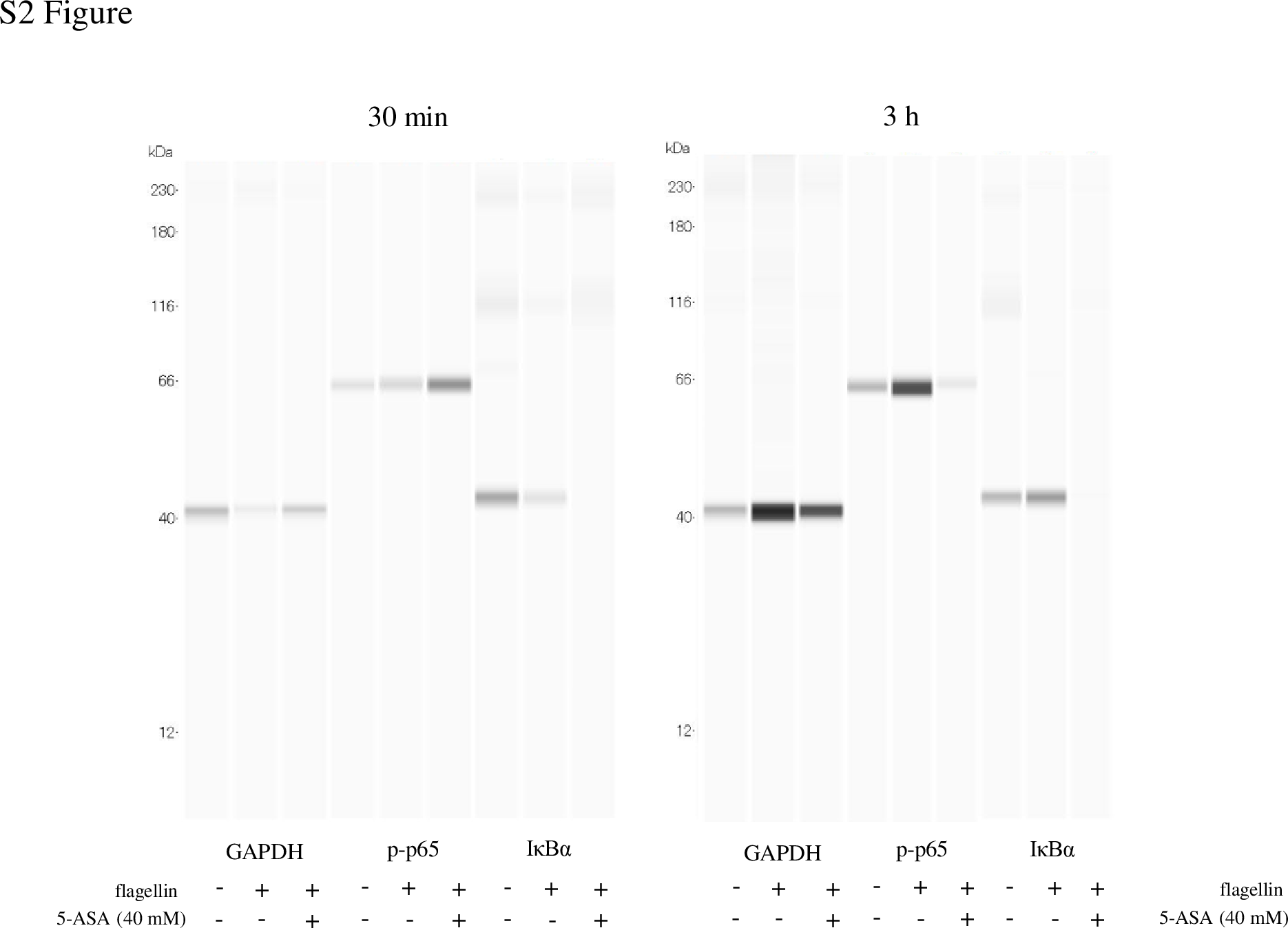

Supplement: S2 Fig — (TIF) [file pone.0264836.s002.tif]
