# Supplementary material for: Promoting mechanism of serum amyloid a family expression in mouse intestinal epithelial cells
Source: PLoS One. 2022 Mar 18;17(3):e0264836. doi: 10.1371/journal.pone.0264836 (PMC8932556; doi:10.1371/journal.pone.0264836)
Supplement: Attachment — Submitted filename: Response_to_Reviews.docx [file pone.0264836.s003.docx]

November 26, 2021
Wendy Huang, Ph.D.
Academic Editor
PLOS ONE

Dear Editor and reviewers:

Thank you for reviewing our paper. We have responded to your comments below and have made corresponding changes to the manuscript. The manuscript has benefited from your insightful suggestions. We hope that the revised manuscript is now suitable for publication in the journal.

Sincerely,

Masaki Wakai, M.D.

Department of Gastroenterology and Metabolism, Hiroshima University Hospital

1-2-3 Kasumi, Minami-ku, Hiroshima 734-8551, Japan

Phone: +81-82-257-5939; Fax: +81-82-257-5939

E-mail: [d175100@hiroshima-u.ac.jp](mailto:d175100@hiroshima-u.ac.jp)

Sincerely,

Ryohei Hayashi, M.D., Ph.D.

Department of Endoscopy, Hiroshima University Hospital

1-2-3 Kasumi, Minami-ku

Hiroshima 734-8551

Japan

Telephone: +81-82-257-5939

Fax: +81-82-257-5939

E-mail: r-hayashi@hiroshima-u.ac.jp

In Figure 1, I changed the description of ascending colon to proximal colon.

Reviewer #1:

Major
I would like to know that the experiment was repeated, especially DSS colitis. If it was repeated, please mention that in the text (Materials and Methods). The error bars of SD was relatively big. Showing each value as a dot must be the best approach for all the readers. Please show all the RT-PCR data in the graphs using dot plots.

Reply: Thank you for your valuable suggestions. The DSS-induced colitis model experiment was performed in two replicates and was found to be reproducible. It is now described in the DSS-induced colitis model section of Materials and Methods (page 5, line 101–102). In addition, the RT-PCR data has been edited so that each value is displayed as a dot.

Please explain how the authors determined the concentration of these cytokines for the detection of SAAs. Basically, the authors should have had several titrations (dilution) to optimize the experiment. The concentration which showed saturated expression of SAA must not be used.

Reply: Thank you for your valuable feedback. The concentration was set by referring to previous papers on cell stimulation. Since there was no difference in the expression of SAA when stimulated with IL-1β and IL-23, we examined the expression of SAA with these cytokines in a concentration-dependent manner (there was a report that SAA increased when stimulated with IL-1β in experiments using cancer cells), and found no significant difference in the enhancement of SAA at any concentration.

In this study, the best option was to use human organoids to check the SAAs expression; even the authors used the DSS animal model in Figure 1. Please consider doing this experiment. So far, only using cell line and mouse organoid are very weak.

Reply: Thank you for your excellent suggestion. As you pointed out, I also think it would be very meaningful to use human organoids to study the response to cytokines. However, using human samples would require a new ethics review which would take a substantial amount of time, so we decided to save this for future experiments.

The biggest concern (issue) in this paper was the analyzing method for the RT-PCR data. What does “normalized SAA expression” mean? All the data looks like a relative expression somewhere. Each data sets have 1.0 in somewhere. Did the author set one data set 1.0, and then relative levels were presented? In the method section, it was mentioned that samples were normalized by (must be by) ACTB expression in each organ and organoid). This was the right way. However, the expression of SAAs was quite low. For example, if you have just 0.00001 for SAA1 in untreated, then they should not use the relative expression. If the quite low value was set as 1.0, then the obtaining relative expression value must be really high, and all the samples are messed up and cannot be compared. Based on this point, all the data did not completely make sense. The authors must learn this and re-analyze all the RT-PCR data correctly.
Reply: Thank you for your suggestions. The graph of PCR results was replaced with a dot plot showing the actual measured values (SAA1-4 divided by ACTB).

Minor:
It would be interesting if the author shows the expression levels of cytokine receptors using organoids (ideally both mouse and human organoids).

Reply: As you pointed out, it would be interesting to examine the expression level of cytokine receptors, but this paper was not focused on the analysis of receptors. Also, since mRNA expression was observed, we believe that the receptors were responding. A paper showing that IL-22-R is expressed is given below:

"IL-22BP dictates characteristics of Peyer’s patch follicle-associated epithelium for antigen uptake", *Journal of Experimental Medicine*, Toshi Jinnohara et al. doi: [10.1084/jem.20160770](http://dx.doi.org/10.1084/jem.20160770)

Please show fluorescent minus one for the immunohistochemistry data for Figures 3C and 4B)

Reply: Although it was not presented in the figure, the images were taken using appropriate negative control methods to avoid autofluorescence. This is also described in the Materials and Methods; Immunofluorescence staining section (page 8, line 144–146) and figure 3 and 4 legends (page 13, line 265 –266; page 14, line 305-306). I have also attached the image below.

Minus one of Figure3C

Minus one of Figure4C

Please provide the information about 5-ASA. Need to explain in more detail why the authors used this drug? What are the differences between NFkb inhibitors (BAY11-7082) and 5-ASA? The author mentioned that In previous reports, NF-κB was suppressed by 20–40 mM 5-ASA in HCT-116 and Caco-2 human colon cancer cell lines. Then what was the purpose of the use of 5-ASA? It sounds just like a very similar experiment.

Reply: Thank you for pointing this out. 5-ASA is the most widely used drug in the treatment of UC. There are multiple pharmacological effects of 5-ASA, including NFκB inhibition. Since 5-ASA exerts a local anti-inflammatory effect in UC, we wanted to investigate whether it acts on the intestinal epithelium itself. Furthermore, past studies have been conducted on cancer cell lines, and it is not certain whether the same physiological reactions occur in normal cells. Therefore, we wanted to examine effects in organoids, which are normal cells. Also, although there have been reports of experiments in which 5-ASA inhibits NFκB, no paper has shown that SAA is inhibited. We believe that this paper is novel in this respect.

Reviewer #2:

1) In Figure 3, the authors showed both LPS (through TLR4) and flagellin (through TLR 5) induced SAA1 and 3’s mRNA expression. However, the NF-kB inhibitor BAY11-7082 treatment was only done in flagellin (Fig 3B) but LPS (Fig 3A) stimulation. LPS is known to activate both canonical and non-canonical NF-kB signaling. Do the authors have any idea/data suggesting with pathways it is?

Reply: Thank you for your suggestions. Since FLA and LPS are in the same TLR pathway, we thought that we would get similar results and thus did not conduct experiments using NFκB inhibitors. In response to your suggestion, we conducted an experiment to see if SAA was suppressed by NFκB inhibitors during LPS stimulation, but contrary to our expectation, we did not find this trend. When we researched past papers to elucidate the reasons behind this, we could not find a sufficient explanation.

2) After pre-treatment with BAY11-7082, the NF-kB activities was not monitored. BAY11-7082 was known to inhibit IkBalpha phosphorylation, as well as directly inhibit functions on the NLRP3 inflammasome by blocking the sensor's ATPase activity. Therefore, showing the IkBa’s protein degradation and NF-kB nuclear translocation is necessary to support the inhibitory effects of SAA expression was through NF-kB signaling.

Reply: Thank you for your suggestion. We believe that BAY11-7082 inhibits NF-κB. We confirmed that inhibition of protein degradation in IκB (phosphorylation of IκB was suppressed) by BAY11-7082 via western blotting.

After this suggestion, we thought it would be interesting to perform the experiment for 5-ASA as well. We have added these results to the Results section (page 13, line 273–283).

3)If the expression of SAA is regulated by NF-kB, is SAA a direct target gene of NF-kB or it is a secondary effect of NF-kB activation? If it is a direct target gene, what is the kB binding site(s)?
Reply: This is an interesting question. We think that SAA is a direct target gene target of NF-κB. As shown in the previous report below, the NFκB binding region (5-GGGACTTTCC-3) exists in the enhancer region of the SAA gene. We have added this to the discussion (page 16, line 346–349).

Li XX, Liao WS. Expression of rat serum amyloid A1 gene involves both C/EBP-like and NF kappa B-like transcription factors. Journal of Biological Chemistry. 1991;266: 15192-15201. doi: 10.1016/S0021-9258(18)98603-5

4) The authors only showed SAA mRNA expression level upon different cytokines and TLR ligands stimulation, how about the SAA proteins’ expression level? It will be interest to see the protein level as well.

Reply: Thanks for the valuable suggestion. As you pointed out, this is a very interesting point, but we have confirmed that SAA expression is increased by immunostaining, and we think that the protein expression level is also increased.

5) In general, all the gene expression figures have huge error, especially Figure 1 and 2; some panels the error bar is larger than the bar graph itself. Figure 1A does not have p-value either. Box & Whisker plots with overlay of individual data points are needed to show the significance of the data.

Reply: Thank you for pointing this out. The graph of PCR results was replaced with a dot plot showing the actual measured values (SAA1-4 divided by ACTB).
