# Supplementary material for: Promoting mechanism of serum amyloid a family expression in mouse intestinal epithelial cells
Source: PLoS One. 2022 Mar 18;17(3):e0264836. doi: 10.1371/journal.pone.0264836 (PMC8932556; doi:10.1371/journal.pone.0264836)
Supplement: Attachment — Submitted filename: Response_to_Reviews.docx [file pone.0264836.s004.docx]

Please review your reference list to ensure that it is complete and correct. If you have cited papers that have been retracted, please include the rationale for doing so in the manuscript text, or remove these references and replace them with relevant current references. Any changes to the reference list should be mentioned in the rebuttal letter that accompanies your revised manuscript. If you need to cite a retracted article, indicate the article’s retracted status in the References list and also include a citation and full reference for the retraction notice.

Reply:

We have confirmed that the reference list is complete and accurate.

In response to Reviewer #1, point #2, the authors stated that “We examined the expression of SAA with these cytokines in a concentration-dependent manner… and found no significant difference”. No data figure were shown. The authors should at least include in the revised Result text section the exact range of the concentrations tested that yield the negative result. For example, “IL-1β (1, 100, 1000 ng/mL) and IL-23 (5, 50, 500 ng/mL) were also tested and similarly returned no significant induction of SAA in the intestinal organoid culture.”

Reply:

Thank you very much for your valuable suggestion. We have added the following text to the Results section (Page 11, Lines 222–224):

“IL-1β (1, 50, 100 ng/mL) and IL-23 (10, 50, 100 ng/mL) levels were also tested and similarly showed no significant induction of SAA in intestinal organoid cultures.”

Related to Reviewer #2, point #1: On page 12 of the text, the text stated “Figure 3. Flagellin and lipopolysaccharide (LPS) promote SAA1-4 expression in small intestinal organoid via NF-kB”. However, Fig 3 only included NF-kB inhibition results in the Flagellin condition, but not the LPS treatment. Therefore, Reviewer #2 asked NF-kB inhibitor to be tested on LPS treated cells. As the authors noted on page 59 of the point-by-point, result of this additional experiment suggest LPS induced SAA expression is NF-kB independent (data not shown). Given this new information, the authors should consider moving the Figure 3A(LPS) panel to a separate figure and take “LPS” out of the Figure 3 title/legend etc – the current writing would give readers the wrong impression that LPS induced SAA is dependent on NFkB. Instead, LPS+NFkB inhibitor results should be shown under a separate figure - texts in the results section should include description of the negative result (e.g. In contrast to the flagellin pathway, LPS induced SAA expression is NF-kB independent.). Discussion should be revised to comment on potential non-NFkB dependent pathway that maybe involved in LPS induced SAA expression subject to future studies.

Reply:

We agree with the pertinent comments made by you. In accordance with your suggestion, the two graphs showing the change in SAA expression with LPS have been separated into separate figures (Supplement Fig 1A and 1B). We have also removed the word “LPS” from the title of Figure 3. In contrast to the flagellin pathway, the induction of SAA expression by LPS is NF-κB-independent; we have mentioned this in the Results section (Page 12, Lines 259–260). A mention of the need for further research to decipher the non-NF-κB-dependent pathway that may be involved in the induction of SAA expression by LPS has been made in the Discussion section (Page 17, Lines 367–368.)

Related to the author’s response to Reviewer #2, point #2: For Figure 4B, western experiment missing loading control (e.g. beta-actin or GAPDH). Figure legend needs to state how many time this experiment had been performed independently showing similar results. Original un-cropped gel images/scans should be displayed.

Reply:

Thank you for your valuable feedback. Figure 4B shows panels for GAPDH expression, used as a control. We have also mentioned in the text that this experiment was repeated twice and that the same trend was observed (Page 14, Line 309–310). The original, untrimmed gel image is shown in Supplement Figure 2, and the figure has been referred to in the manuscript (Page 14-15, Lines 310–311).
